# Adams County Medical Society

**Published:** 1867-09

**Authors:** Joseph Robbins

**Affiliations:** Secretary


### Proceedings of Societies.

### ADAMS COUNTY MEDICAL SOCIETY.

The regular annual meeting of the Adams County Medical Society assembled at the office of Dr. Robbins, Quincy, May 13, 1867, at 10½ o'clock A.M., the President, Dr. J. N. Ralston, in the Chair.

Present—Drs. Ralston, Helms, Watson, Wilson, Kendall, Bassett, R. Williams, Baker, Drude, Cadwell, Martin, and Robbins.

The minutes of the last regular, and subsequent called meetings, were read and approved.

The treasurer, Dr. Watson, presented his annual report, which was read and ordered on file.

Dr. Kendall moved that the charges against Dr. M. F. Bassett be taken up for consideration. Pending which the President explained why he had no annual address prepared; and, on motion of Dr. Drude, the Society adjourned to 2 o'clock P.M.

AFTERNOON SESSION.

The Society was called to order at 2 o'clock P.M., the President, Dr. Ralston, in the Chair.

Present—Drs. Ralston, Bane, Watson, Moss, Bassett, Martin, Helms, McNeall, W. J. Brown, C. C. Brown, Wilson, Williams, Drude, Morris, Baker, Kendall, Lunn, Cadwell, and Robbins.

The motion to take up the case of Dr. Bassett, pending when the Society adjourned, was carried.

Dr. Bassett being called upon, responded that he was ready to proceed with the case, and proceeded to state that the reasons given in the circular letter which he had issued to the profession, were those that impelled him to the course he had pursued. He had used the remedy there referred to in many cases of carcinomatous disease, and in none without benefit. Serious illness had interfered with his investigations, and it was too early to estimate the true value of the remedy. He had submitted it to a competent chemist, who had succeeded in ascertaining some of its constituents. These results he would give to the profession, and could he succeed in learning all the constituents, he would give it all to them. He had had no concealment, and desired none. He plead "not guilty."

The circular issued by Dr. Bassett was then read.

Dr. Watson moved an acquittal of the charge contained in the first specification, which was carried by the following vote:

*Ayes*—Ealston, Watson, Wilson, Drude, Robbins, Baker, Williams, McNeall, Martin, Moss, Morris.

*Nayes*—Kendall, Helms, Cadwell, Bane.

Dr. Robbins moved an acquittal on the second specification.

After considerable discussion and further remarks by Dr. Bassett, Dr. Bane offered as a substitute for the motion of Dr. Robbins, the following:

*Whereas*, On the 10th of December, 1866, charges were preferred aginst Dr. M. F. Bassett, of having violated ethical principles contained in chapter ii., sections 3 and 4, of the Code of Medical Ethics of the American Medical Association; and,

*Whereas*, He has this day satisfactorily explained the course on which such charges were predicated, stating that he fully believed himself to be acting within the scope of the code of ethics; therefore

*Resolved*, That said charges and specifications be indefinitely postponed.

On motion of Dr. Robbins, the resolution was amended, by striking out the words "*indefinitely postponed*," and inserting in lieu thereof the word "*dismissed*."

On motion of Dr. Bane, the second clause of the preamble was amended by inserting after the word "ethics," the following, viz.: "admitting a technical violation of the letter of section 4 of said code, but not of its spirit."

The preamble and resolutions, as amended, was then adopted by the following vote:

*Ayes*—Watson, Wilson, Drude, Robbins, Moss, Williams, Martin, Morris.

*Nays*—Ralston, Kendall, Helms, Cadwell, Bane, Brown, McNeall.

Dr. Robbins moved a reconsideration, which motion to reconsider was, on his own motion, laid on the table.

Charles C. Brown, M.D., was proposed for membership by his father, Dr. Wm. J. Brown, and the proposition referred to the Censors, who reported him a regularly qualified physician, in good standing, he having graduated at Rush Medical College, Chicago, his diploma bearing date January 25, 1867.

On motion, he was regularly admitted to membership.

On the application of Dr. C. R. S. Curtis for membership, the Censors report him a regularly qualified physician, a graduate of the New York Medical College, his diploma bearing date February, 1854.

The report was referred back to the Censors, with instructions to confer with Dr. Curtis, relative to some publications in the daily papers, supposed to have been made with his knowledge, and consult and report at the next regular meeting.

On motion, the regular order of business was suspended, and the Society proceeded to the election of officers for the ensuing year.

The following named gentlemen were elected:

*President*—Dr. Louis Watson.

*Vice-Presidents*—Dr. J. W. Bartlett, Dr. M. M. Bane.

*Recording and Corresponding Secretary*—Dr. J. Robbins.

*Treasurer*—Dr. H. W. Kendall.

*Censors*—Dr. J. T. Wilson, Dr. M. F. Bassett, Dr. Richard Williams.

*Ordered*, That the dues of members who have retired from active practice be remitted, and that they be exempt from paying dues until otherwise ordered.

Dr. Wilson, one of the essayists for this meeting, reported a paper prepared, but, owing to the lateness of the hour, its hearing was postponed until the next regular meeting.

Drs. Landon and Roeschlant both being absent, and it being understood that both had papers prepared, their appointment as essayists was continued.

Drs. Joseph Robbins, Louis Watson, H. W. Kendall, J. T. Wilson, and Richard Williams, were elected delegates to the Illinois State Medical Society, and it was ordered that each delegate be authorized to appoint his own substitute, in case of inability to attend.

Dr. Robbins offered the following resolution, which was adopted:

*Resolved*, That a cordial invitation be, and is, hereby extended to the Illinois State Medical Society, to hold its next annual meeting in the city of Quincy; and we assure its members of a hearty welcome from their professional brethren in the City of Quincy, and the County of Adams. Adjourned.

JOSEPH ROBBINS, M.D., Secretary.

---

MR. EDITOR:—

At the Regular Quarterly Meeting of the Adams County

Medical Society, held in this city on Monday, the 12th ult., it was ordered that such portion of the proceedings of said meeting as referred to the case of Dr. Bassett, be published in the Chicago medical journals.

Under this order I transmit the following:

Dr. Bane moved to take from the table the motion to reconsider the vote by which the resolution referring to the charges against Dr. Bassett, was passed at the Annual Meeting in May.

Dr. Robbins raised a question of order.

After considerable discussion the President decided the motion of Dr. Bane out of order, but invited an appeal.

An appeal was taken by Dr. Bane; the yeas and nays were demanded, and the decision of the Chair reversed by the following vote:

*Ayes*—Wilson, Bassett, Robbins, Williams, Bonney, Martin, 6.

*Nays*—Ralston, Stahl, Bartlett, Kendall, Helms, Baker, Shepherd, Cadwell, Hess, Bane, Landon, Young, Leach, 13.

The yeas and nays were then taken on the motion to reconsider, and it was carried:

*Ayes*—Ralston, Stahl, Bartlett, Kendall, Helms, Baker, Shepherd, Cadwell, Hess, Bane, Landon, Young, Leach, 13.

*Nays*—Watson, Wilson, Robbins, Williams, Bonney, Martin, 6.

On motion of Dr. Young, the charges against Dr. Bassett were referred to a committee of five, with instructions to report at the Annual Meeting in May next, said committee to be appointed by the Society.

The Society appointed Drs. Wilson, Bartlett, Ralston, Robbins, and Bane, to that committee.

Dr. Ralston moved the suspension of Dr. Bassett from the rights and privileges of membership until the next Annual Meeting.

A two-thirds vote being required, the motion was, on taking the yeas and nays, lost by the following vote:

*Ayes*—Ralston, Stahl, Kendall, Helms, Baker, Shepherd, Cadwell, Hess, Bane, Landon, Leach, 11.

*Nays*—Watson, Bartlett, Wilson, Robbins, Young, Williams, 6.

A true copy from the records.

JOSEPH ROBBINS, Secretary.

